# Scalable Data Mining Algorithms in Computational Biology and Biomedicine

**DOI:** 10.1155/2017/5652041

**Published:** 2017-02-28

**Authors:** Quan Zou, Dariusz Mrozek, Qin Ma, Yungang Xu

**Affiliations:** ^1^School of Computer Science and Technology, Tianjin University, Tianjin 300354, China; ^2^Institute of Informatics, Silesian University of Technology, 44-100 Gliwice, Poland; ^3^Department of Mathematics and Statistics and Department of Agronomy, Horticulture, and Plant Science, BioSNTR, South Dakota State University, Brookings, SD 57007, USA; ^4^Center for Bioinformatics and Systems Biology, Wake Forest School of Medicine, Winston-Salem, NC 27157, USA

Since “Precision Medicine” was initially launched by President Obama, it presents a huge challenge and chance for the computational biology and biomedicine. In recent years, computational methods appeared vastly in the biomedicine and bioinformatics research, including medical image analysis, healthcare informatics, and cancer genomics. Lots of prediction and mining works were required on the medical data, such as tumor images, electronic medical records, microarray, and GWAS (Genome-Wide Association Study) data. Therefore, a growing number of data mining algorithms were employed in the prediction tasks of computational biology and biomedicine.

Advanced data mining techniques have also been developed quickly in recent years. Several impacted new methods were reported in the top journals and conferences. For example, affinity propagation was published in Science as a novel clustering algorithm. Recently, deep learning seems to be suitable for big data and is becoming the next hot topic. Parallel mechanism is also developed by the scholar and industry researchers, such as Mahout. A growing number of computer scientists are devoted to the advanced large scale data mining techniques. However, application in biomedicine has not fully been addressed and fell behind the technique growth.

This special issue targeted the recent large scale data mining techniques together with biomedicine application and provided a platform for researchers to exchange their innovative ideas and real biomedical data. We have received 25 manuscripts from Asia, Europe, and America, of which 21 papers were accepted. We categorize three subtopics for our special issue.

The first part contains 5 papers that are related to biomedicine images. The paper “Convolutional Deep Belief Networks for Single-Cell/Object Tracking in Computational Biology and Computer Vision” proposed a convolutional deep belief network based architecture to dynamically learn the most discriminative features from data for both single-cell and object tracking in computational biology, cell biology, and computer vision. The paper “Objective Ventricle Segmentation in Brain CT with Ischemic Stroke Based on Anatomical Knowledge” proposed detection system of ischemic stroke in CT, which can exclude the stroke regions from segmentation result with a combined segmentation strategy. The paper “Segmentation of MRI Brain Images with an Improved Harmony Searching Algorithm” proposed a modified algorithm to improve the efficiency of the algorithm. First, a rough set algorithm was employed to improve the convergence and accuracy of the HS algorithm. Then, the optimal value was obtained using the improved HS algorithm. The optimal value of convergence was employed as the initial value of the fuzzy clustering algorithm for segmenting magnetic resonance imaging (MRI) brain images. The paper “Functional Region Annotation of Liver CT Image Based on Vascular Tree” proposed a vessel-tree-based liver annotation method for CT images based on the topological graph. A hierarchical vascular tree is constructed to divide the liver into eight segments according to Couinaud classification theory and thereby annotate the functional regions. The paper “Robust Individual-Cell/Object Tracking via PCANet Deep Network in Biomedicine and Computer Vision” proposed a robust feature learning method for robust individual-cell/object tracking, which constructed a discriminative appearance model via a PCANet deep network without large scale pretraining.

The second part contains 12 papers on bioinformatics. The paper “A Metric on the Space of Partly Reduced Phylogenetic Networks” proposed a polynomial-time computable metric on the space of partly reduced phylogenetic networks based on the equivalent nodes, whose space is much closer to the space of rooted phylogenetic networks than the others. The paper “Statistical Approaches for the Construction and Interpretation of Human Protein-Protein Interaction Network” established a reliable human protein-protein interaction network and developed computational tools to characterize a protein-protein interaction (PPI) network, where confidence measures were assigned to each derived interacting pair and account for the confidence in the network analysis. The paper “*In Silico* Prediction of Gamma-Aminobutyric Acid Type-A Receptors Using Novel Machine-Learning-Based SVM and GBDT Approaches” proposed a machine-learning-based method for GABAARs prediction at high and low identity data, which sufficiently captured features only from the protein (GABAARs and non-GABAARs) sequence information based on 188-dimensional algorithm and made predictions by Gradient Boosting Decision Tree, Random Forest, libSVM, and *k*-NN classifiers. By integrating gene expression and DNA methylation data, the paper “Uncovering Driver DNA Methylation Events in Nonsmoking Early Stage Lung Adenocarcinoma” proposed a bioinformatics pipeline of differential network analysis to uncover driver methylation genes and responsive DNA methylation-mediated modules contributing to tumorigenesis. The computational pipeline successfully identified driver epigenetic events in nonsmoking early stage of lung adenocarcinoma. The paper “Identification of Bacterial Cell Wall Lyases via Pseudo Amino Acid Composition” proposed a computational method for Bacterial Cell Wall Lyase prediction, which can obtain optimal features from pseudo amino acid composition by using ANOVA-based feature selection technique. The paper “Recombination Hotspot/Coldspot Identification Combining Three Different Pseudocomponents via an Ensemble Learning Approach” proposed a new computational predictor for recombination hotspot identification only based on the DNA sequences, which combined three kinds of features via an ensemble learning technique. It would be a useful tool for DNA sequence analysis. The paper “Analysis of Important Gene Ontology Terms and Biological Pathways Related to Pancreatic Cancer” investigated the pancreatic cancer by extracting important related GO terms and KEGG pathways. The enrichment theory of GO and KEGG pathway was adopted to encode the validated genes and other genes. And the mRMR method was used to analyze the importance of each GO term and KEGG pathway. Furthermore, the obtained GO terms and KEGG pathways were extensively analyzed. The paper “Constructing Phylogenetic Networks Based on the Isomorphism of Datasets” researched the commonness of the methods based on the incompatible graph, the relationship between incompatible graph and the phylogenetic network, and the topologies of incompatible graphs. The paper “A Novel Peptide Binding Prediction Approach for HLA-DR Molecule Based on Sequence and Structural Information” proposed a novel prediction method for MHC II molecules binding peptides, which calculated sequence similarity and structural similarity between different MHC II molecules and produced a combined similarity score to predict binding cores. The paper “ProFold: Protein Fold Classification with Additional Structural Features and a Novel Ensemble Classifier” proposed a machine learning method for protein fold classification, which imports protein tertiary structure in the period of feature extraction and employs a novel ensemble strategy in the period of classifier training. The paper “An Approach for Predicting Essential Genes Using Multiple Homology Mapping and Machine Learning Algorithms” aimed to test whether optimizing the weight coefficients by the machine learning method could improve the accuracy of their previously proposed evolutionary feature based model, which had shown the best prediction among all published algorithms for predicting bacterial essential genes, and finally the adaption achieved a small improvement. The paper “Positive-Unlabeled Learning for Pupylation Sites Prediction” employed PU learning for predicting pupylation sites and got better performance than traditional classifiers.

The third part contains 4 papers and focuses on the computational medicine. The paper “Optimization to the Culture Conditions for Phellinus Production with Regression Analysis and Gene-Set Based Genetic Algorithm” proposed an optimal method for Phellinus production, where regression model was obtained by sampling data and gene-set based genetic algorithm was applied to find optimized factors for Phellinus production. The paper “Depth Attenuation Degree Based Visualization for Cardiac Ischemic Electrophysiological Feature Exploration” implemented a human cardiac ischemic model and revealed the hidden cardiac biophysical behavior under the ischemic condition by the depth attention degree based optic attenuation model, which effectively explored the important features of interest of the heart under the pathological condition with complex electrophysiological context and is fundamental in analyzing and explaining biophysical mechanisms of cardiac functions for the doctors and medical staffs. The paper “Analysis and Classification of Stride Patterns Associated with Children Development Using Gait Signal Dynamics Parameters and Ensemble Learning Algorithms” computed the sample entropy and average stride interval parameters to quantify the gait dynamics of children with different age groups and used the AdaBoost.M2 and Bagging ensemble learning algorithms to effectively perform gait pattern classifications. The paper “A Computational Method for Optimizing Experimental Environments for* Phellinus igniarius* via Genetic Algorithm and BP Neural Network” used training data to build a neural network model, which acts as the fitness function for further optimal condition finding with genetic algorithm.

To conclude, papers in this special issue cover several emerging topics of scalable data mining techniques and applications for biomedicine or bioinformatics. We highly hope this special issue can attract concentrated attentions in the related fields.

